# Supplementary figures and images for: A Non-Canonical Pathway Induced by Externally Applied Virus-Specific dsRNA in Potato Plants
Source: Int J Mol Sci. 2023 Oct 30;24(21):15769. doi: 10.3390/ijms242115769 (PMC10650801; doi:10.3390/ijms242115769)

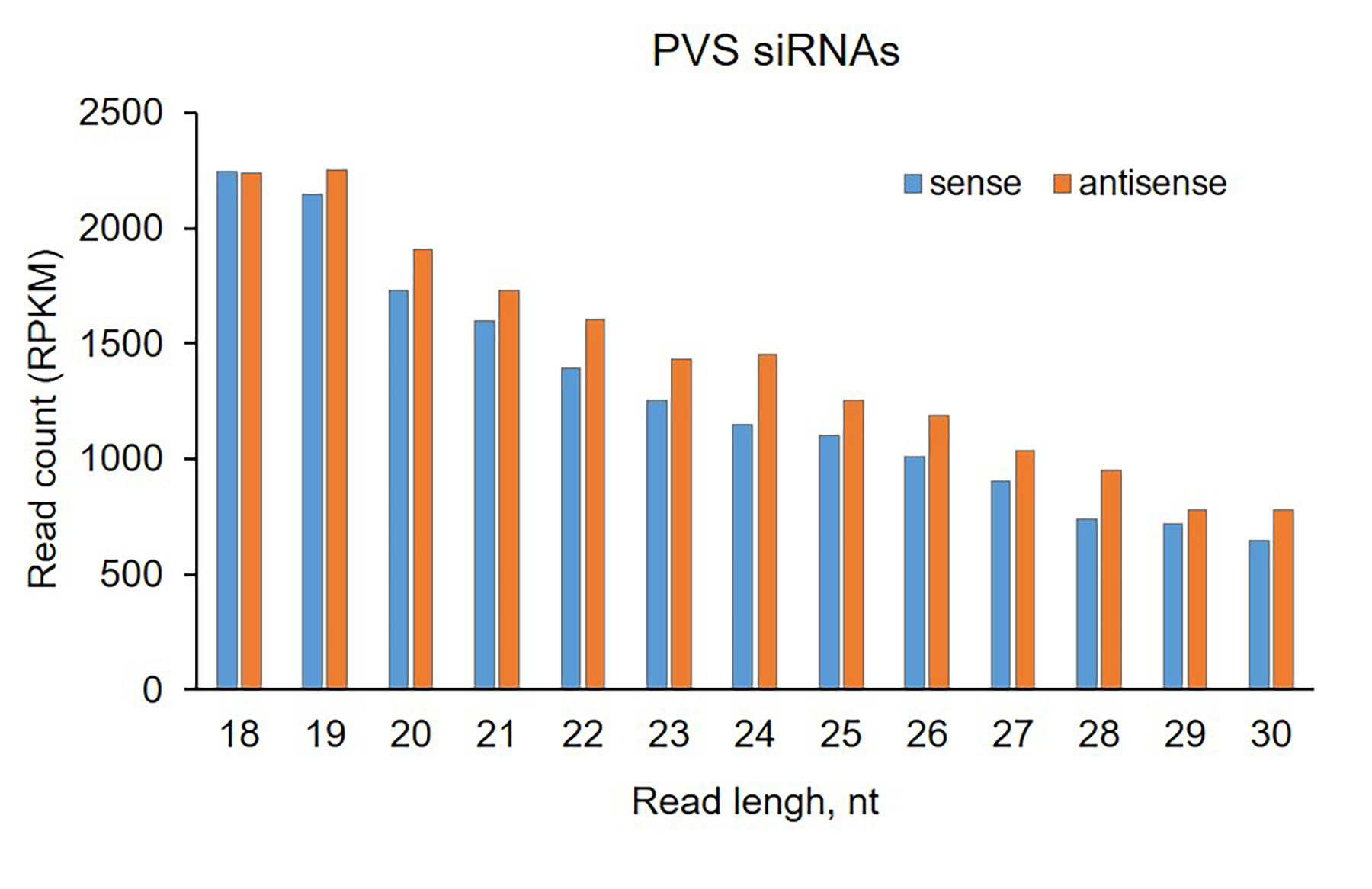

Supplement: Supplementary file 1 [file ijms-24-15769-s001.zip › Figure S1.jpg]

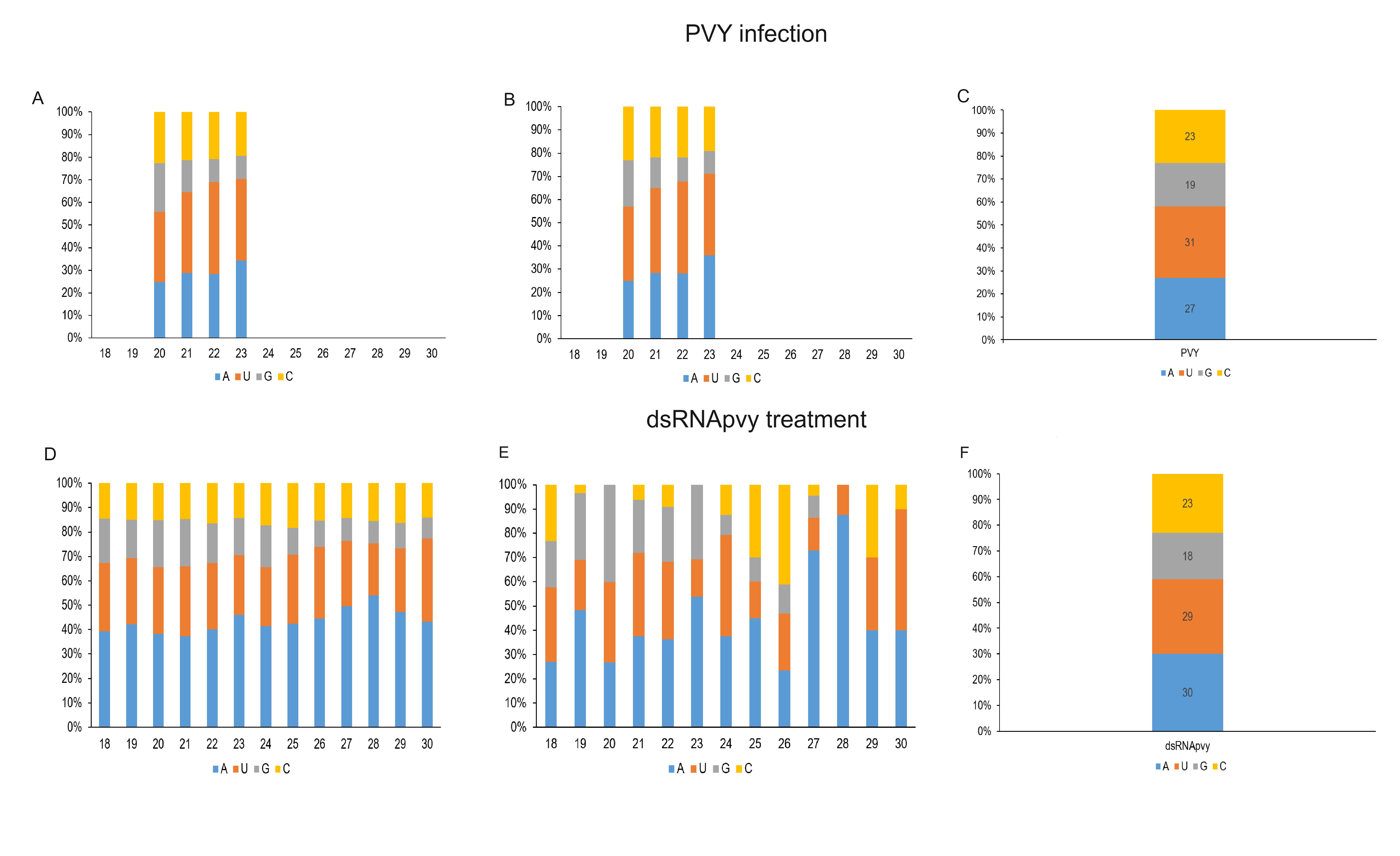

Supplement: Supplementary file 1 [file ijms-24-15769-s001.zip › Figure_S2.jpg]
